# Association between human papillomaviruses, metabolic syndrome, and all-cause death; analysis of the U.S. NHANES 2003–2004 to 2015–2016

**DOI:** 10.1371/journal.pone.0299479

**Published:** 2024-03-07

**Authors:** Parmis Mirzadeh, Akinkunle Oye-Somefun, Chris I. Ardern, Catriona J. Buick

**Affiliations:** 1 School of Kinesiology and Health Science, Faculty of Health, York University, Toronto, Canada; 2 School of Nursing, Faculty of Health, York University, Toronto, Canada; 3 Odette Cancer Centre, Sunnybrook Health Sciences Centre, Toronto, Canada; Katholieke Universiteit Leuven Rega Institute for Medical Research, BELGIUM

## Abstract

**Introduction:**

Human papillomavirus (HPV) is the most common sexually transmitted infection, attributed to 4.5% of all cancers worldwide. Co-infection with the metabolic syndrome (MetS), a common cluster of cardiometabolic risk factors, has been shown to increase the persistence of HPV. The purpose of this study was to estimate the association between HPV and MetS on mortality risk.

**Methods:**

Data for the current study was drawn from seven consecutive cycles (2003–2004 to 2015–2016) of the U.S. NHANES. The final analytic sample consisted of 5,101 individuals aged 18-65y with HPV and MetS information with follow-up to Dec. 31^st^, 2019. Baseline HPV status was assessed by either vaginal swab, penile swab or oral rinse and used to classify participants as: no HPV (n = 1,619), low (n = 1,138), probable (n = 672), and high-risk (n = 1,672; 22% type 16, and 10% type 18) HPV using IARC criteria. MetS was assessed by the Harmonized criteria.

**Results:**

The average follow-up was 9.4 y with 240 all-cause deaths (no HPV: n = 46 deaths; low-risk: n = 60 deaths; probable: n = 37 deaths, and; high-risk: n = 97 deaths). HPV status alone revealed no associations with mortality in fully adjusted models. Cross-classification into discrete MetS/HPV strata yielded an increased risk of mortality in females with high-risk HPV/MetS relative to the no MetS/no HPV group.

**Conclusions:**

In this study, low, probable, and high-risk HPV and MetS were differentially related to mortality risk in men and women. Further work is necessary to separate the temporal, age, vaccination, and sex effects of HPV diagnosis in these relationships using prospective studies with detailed histories of HPV infection and persistence.

## Introduction

Human Papilloma Virus (HPV) is a common sexually transmitted infection (STI), and the most prevalent STI in the United States, with over 20 million people living with HPV and 5.5 million new cases each year [1]. As of now, over 200 types of human papillomaviruses (HPVs) have been identified, with approximately 40 of them known to infect the genital tract [2]. These include both “low-risk” or “high-risk” HPV subtypes that have been classified based on their association with cancer [3–5]. Low-risk types of HPV can cause genital warts and low-grade intraepithelial neoplasia on the cells of the cervix [6]. High-risk HPV can cause low-grade *and* high-grade intraepithelial neoplasia and are implicated in cancer [6]. Persistent infection by high-risk or oncogenic HPV types is firmly established as the necessary cause of most premalignant and malignant epithelial lesions of the cervix, and a variable fraction of neoplastic lesions of the vulva, vagina, anus, penis, and oropharynx [1, 7]. Of note, high-risk HPV is the cause of 5% of all cancers worldwide [8], and two of most common oncogenic types (HPV 16 and 18) are responsible for ~70% of all cervical cancers [9].

To date, most research on HPV risk has focused on psychosocial predictors, preventive screening, and health system-related factors, with relatively few studies addressing issues of chronic disease comorbidity. Despite an increasing trend in the incidence and mortality associated with cervical cancer over time [10], non-communicable diseases account for the overwhelming burden of premature death worldwide [11], highlighting a need to jointly address factors that may contribute to augmented cervical cancer risk. One such factor is the metabolic syndrome (MetS), a cluster of cardiometabolic risk factors [12] that increases the risk of CVD and all-cause death [13] and is found in more than 40% of the U.S. population [14].

Of importance, MetS has been recently found to both co-occur with HPV, and increase risk of both HPV persistence [15, 16] and HPV-related cancers in the presence of MetS [17] or its individual components [18–25]. Whereas two prior studies have found an increased risk of mortality due to HPV-related cancers [26, 27], no studies to date have quantified the association between HPV and MetS on risk of death. The purpose of this study is to therefore explore the association between HPV and MetS on mortality risk, using a nationally representative sample from U.S. NHANES.

## Methods

### Database

Data for the study were drawn from the U.S. National Health and Nutrition Examination Survey (NHANES), which is a publicly available program of population-based studies on health and nutritional status of males and females of all ages and ethnicities in the United States [27]. The current analysis combines health history and sociodemographic information, dietary questionnaires, physical laboratory examination, and biospecimen (HPV subtype and cardiometabolic biochemistry) components of NHANES. Ethics approval was obtained from the National Center for Health Statistics Research Ethics Review Board (ERB) for NHANES 1999–2004 (Protocol #98–12), NHANES 2005–2010 (Protocol #2005–06), NHANES 2011–2016 (Protocol #2011–17), and written informed consent was obtained from all participants. This study is an analysis of NHANES publicly available anonymized data (Internet address: https://www.cdc.gov/nchs/nhanes/index.htm), and thus, does not require further ethical review from the York University institutional review board.

### Study sample

**Fig 1:**
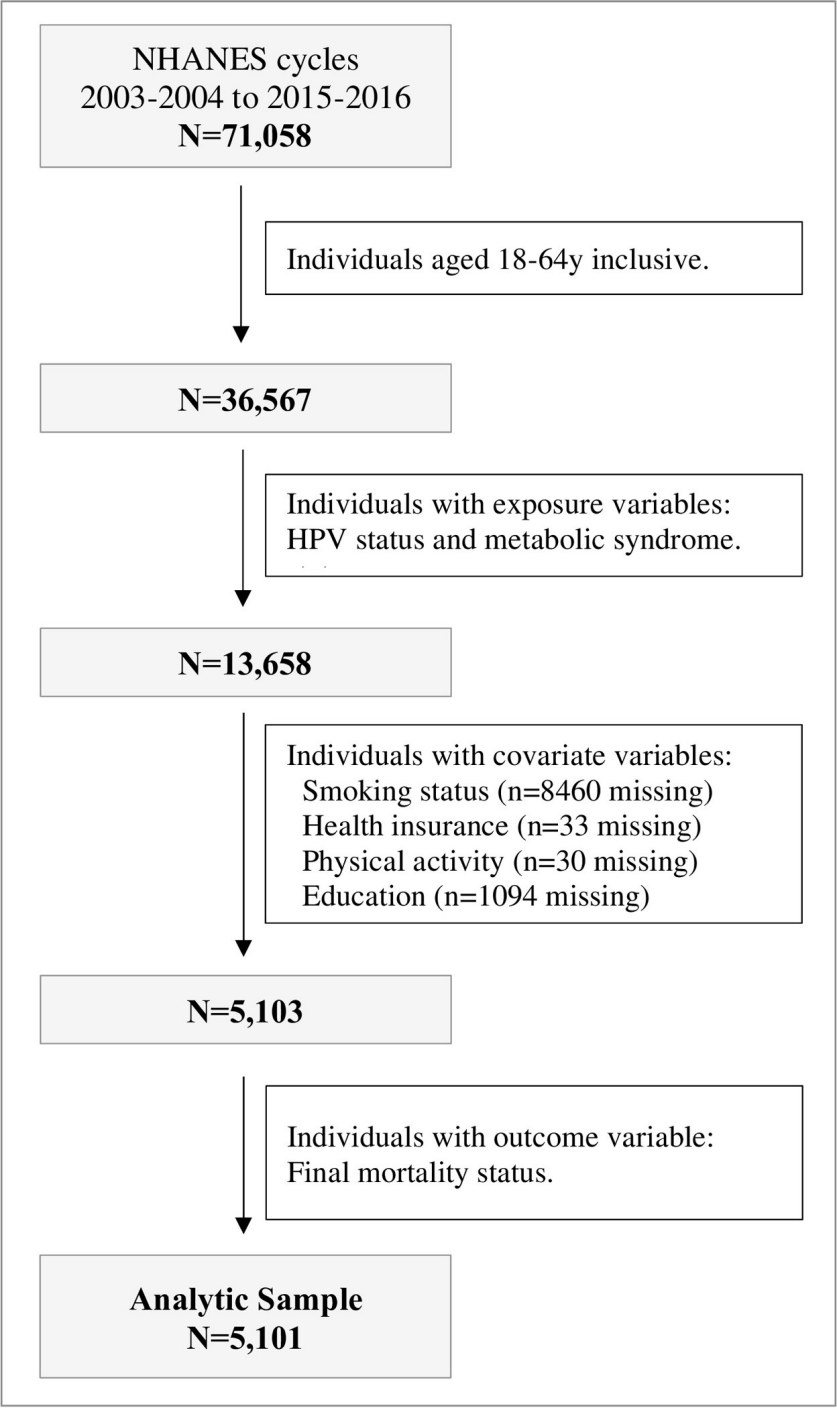
Flow chart of NHANES cycles 2003–2004 to 2015–2016 full sample to final analytic sample.

The present analysis is based on a pooled sample of n = 71,058 participants across seven consecutive NHANES cycles, from 2003–2004 to 2015–2016. The sample was reduced to 36,567 individuals by excluding those under 18 years and above 64 years old, as alterations in body composition can alter assessment of MetS [28]. In total, a subset of n = 13,763 individuals aged 18–64 y had information on HPV and MetS status. After excluding those with missing covariate and follow-up data a final analytic sample of n = 5,101 was available for the current study (**Fig 1**).

### Variables

Baseline HPV status was assessed through vaginal, penile, and oral swabs and classified as positive or negative for each HPV subtype. HPV testing was done through vaginal swabs for females (18-59y, 2003–2004 to 2015–2016) and penile swabs for males (18-59y, 2013–2014 to 2015–2016) using Roche Linear Array Assays, and oral swabs for both males and females (18-69y, 2009–2010 to 2015–2016). Further information on NHANES laboratory methodology is available in the data documentation, codebook, and frequencies files [29]. HPV related cancer risk was subsequently categorized into “no” HPV, “low”, “probable”, and “high” risk HPV groups based on IARC criteria [3]. Individuals with a negative HPV test for all sub-types were categorized into no HPV. Individuals who tested positive for one or more of sub-types 6, 11, 40, 42, 54, 55, 61, 62, 64, 71, 72, 81, 83, 84, or 89 were classified as low-risk; probable-risk was assigned to anyone testing positive for one or more of sub-types 26, 53, 66, 67, 68, 69, 70, 73, or 82, and; anyone testing positive for one or more of sub-types 16, 18, 31, 33, 35, 39, 45, 51, 52, 56, 58, or 59 were classified as having high-risk HPV. As co-infections of different genotypes are common, the low-risk HPV category excluded positive tests for probable or high-risk subtypes, and the probable-risk HPV category excluded positive tests for high-risk subtypes. This was done to ensure that individuals who tested positive for a low-risk and probable or high-risk subtype were not categorized into the low-risk group, and that those with probable-risk and high-risk subtypes were not categorized into the probable-risk groups. In short, individuals with co-infections were coded as the IACR classification with the highest cancer risk; as a result, low-risk sub-types are under-counted in the current analysis.

MetS was defined as having three or more of the following five components: high waist circumference (M: **≥** 102 cm, F: **≥** 88 cm), high blood pressure (systolic **≥** 130 mmHg or diastolic **≥** 85 mmHg, or taking hypertensive medications), high blood glucose (**≥** 100 mg/dl or taking diabetes medication), high blood triglycerides (**≥** 150 mg/dl), or low HDL-cholesterol (M: ≤ 40 mg/dl, F: ≤ 50 mg/dl) [12].

Age, sex, ethnicity, education, health insurance status, smoking status (nicotine), were captured by self-report questionnaire. Height and weight were measured and used to classify body mass index (BMI: kg/m^2^) into categories of “underweight” (BMI < 18.5 kg/m^2^), “healthy weight” (BMI: 18.5–24.9 kg/m^2^), “overweight” (BMI: 25–29.9 kg/m^2^), and “obesity” (BMI ≥ 30 kg/m^2^). Physical activity was assessed by self-reported minutes of activity and coded as “meeting” (150+ min / week) or “not meeting” (< 150 min / week) current recommendations.

### Statistical analysis

Descriptive statistics were used to examine the prevalence of HPV sub-types, stratified by sex. Prevalence of HPV cancer risk groups, demographics, and health behaviors were stratified by sex. A series of multivariable analyses were then developed to account for key sociodemographic and clinical factors. As an intermediate analysis, logistic regressions were performed to estimate the odds of MetS by HPV cancer risk groups using two sex-specific models: 1) unadjusted; 2) adjusted for smoking, age, health insurance, physical activity, and education to prevent confounding effects as these factors may be associated with mortality. Probability of survival across HPV cancer risk groups was subsequently assessed using sex-specific Kaplan Meir curve analysis. Finally, to assess the joint effect of MetS and HPV on mortality risk, these two independent variables were cross-classified into eight discrete groups: i) no HPV and no MetS (HR = 1.0, referent); ii) low-risk HPV and no MetS; iii) probable-risk HPV and no MetS; iv) high-risk HPV and no MetS; v) no HPV and MetS; vi) low-risk HPV and MetS; vii) probable-risk HPV MetS, and; viii) high-risk HPV and MetS. Cox proportional hazard regression analysis was then used to examine the individual and combined effects of HPV and MetS on all-cause mortality in men and women separately (unadjusted; and adjusted for age, smoking status, health insurance, physical activity, and education). The proportional hazards assumption of the log-linear Cox regression model was assessed by a formal test of proportionality with time-dependent cancer-risk groups. This test revealed no violation of proportional hazard assumptions (Wald χ2 = 2.05, df = 2, p = 0.13). Data analysis was performed with SAS software version 9.4. For the analysis of HPV subtype we used unweighted frequencies to visualize the raw frequency distribution. In all other cases except the Kaplan Meier curves, analyses were weighted to be representative of the U.S. population using the svyweight procedure. Statistical significance was set at alpha = 0.05.

## Results

**Table 1** displays the demographic and health characteristics of the sample, stratified by sex (M: 36%; F: 64%) and category of HPV cancer risk (no HPV, low, probable, and high-risk). Young adult females, 18 to 24 years of age, represented half of the population with a high-risk HPV. By contrast, those in the no HPV group were more likely to be non-smokers, individuals with higher education, and young adult males 18 to 24 years of age.

**Table 1:** Characteristics of 5101 U.S. adults aged 18–64 years old, NHANES 2003–2004 to 2015–2016.

	Male					Female				
	36% (n = 1827)					64% (n = 3274)				
	n	No HPV	Low risk	Probable risk	High risk	n	No HPV	Low risk	Probable risk	High risk
		n = 599	n = 392	n = 209	n = 627		n = 1020	n = 476	n = 463	n = 1045
		n = 4003301	n = 2259244	n = 1331405	n = 4063397		n = 7777914	n = 4675956	n = 3120279	n = 6868309
**Age**										
18 to 24 years old	168	41.88	16.41	14.44	27.27	367	20.54	14.24	15.62	49.60
25 to 44 years old	834	37.67	18.23	9.29	34.82	1543	33.07	20.85	13.41	32.66
45 years and older	825	29.17	21.25	12.95	36.62	1364	39.87	22.45	14.01	23.67
**Race/Ethnicity**										
Mexican American	248	44.98	18.71	8.98	27.33	363	36.31	20.36	15.57	27.76
Other Hispanic	188	37.97	24.33	10.23	27.48	258	34.11	22.19	14.14	29.55
Non-Hispanic White	757	33.60	18.06	12.68	35.66	1750	37.03	18.89	13.80	30.28
Non-Hispanic Black	407	19.67	26.25	11.58	42.50	688	19.69	30.95	16.05	33.31
Other	227	46.81	16.53	4.63	32.03	215	32.40	25.71	8.74	33.15
**Education Level**										
Highschool and less	1031	31.95	20.34	11.04	36.67	1631	27.62	21.62	15.98	34.78
Some College/AA degree	537	36.12	17.63	11.40	34.84	1147	35.77	21.22	12.41	30.60
College graduate or above	259	37.77	19.87	12.51	29.84	496	47.70	18.45	12.18	21.67
**Health Insurance**										
Covered	1187	36.30	19.00	11.88	32.82	2378	36.45	20.02	14.22	29.31
Not covered	640	29.45	20.33	10.27	39.96	896	28.75	23.51	12.86	34.87
**BMI Category**										
Underweight	92	36.89	21.30	5.82	35.99	255	26.07	21.99	17.96	33.98
Normal weight	458	34.19	17.87	10.95	36.99	783	35.15	20.11	11.64	33.10
Overweight	675	32.39	19.37	11.73	36.51	848	34.22	19.56	15.08	31.14
Obesity	602	36.18	20.13	12.07	31.61	1388	36.46	22.01	13.81	27.73
**Physical Activity**										
Does not meet guidelines	489	35.27	21.16	9.97	33.61	1422	30.51	23.25	14.19	32.05
Meets guidelines	1338	34.01	18.75	11.94	35.30	1852	37.52	19.16	13.71	29.61
**Smoking (Nicotine) Status**										
Non-smoker	756	41.14	16.37	12.68	29.81	1264	45.94	19.34	11.79	22.93
Smoker	1071	28.32	22.05	10.31	39.32	2010	26.47	21.91	15.44	36.18
**Metabolic Syndrome**										
Yes	415	34.37	20.34	11.90	33.39	853	37.58	22.88	13.16	26.37
No	1412	34.33	19.06	11.26	35.35	2421	33.74	20.20	14.13	31.92
**Mortality Status**										
Alive	1739	35.26	19.38	11.42	33.95	3122	35.13	20.72	13.77	30.37
Dead	88	15.14	19.49	11.54	53.83	152	24.12	23.28	16.88	35.72

This table contains sex stratified weighted frequencies across cancer risk groups to better understand sample characteristics.

Sample sizes (n) across cancer risk groups represent unweighted and weighted values.

Numbers represent percentages across rows.

Sex stratified chi-squared analyses were performed using case counts to assess overall differences across groups and revealed significant differences (p<0.05) across all variables except BMI categories and physical activity in males, and BMI categories and mortality status in females.

**Fig 2:**
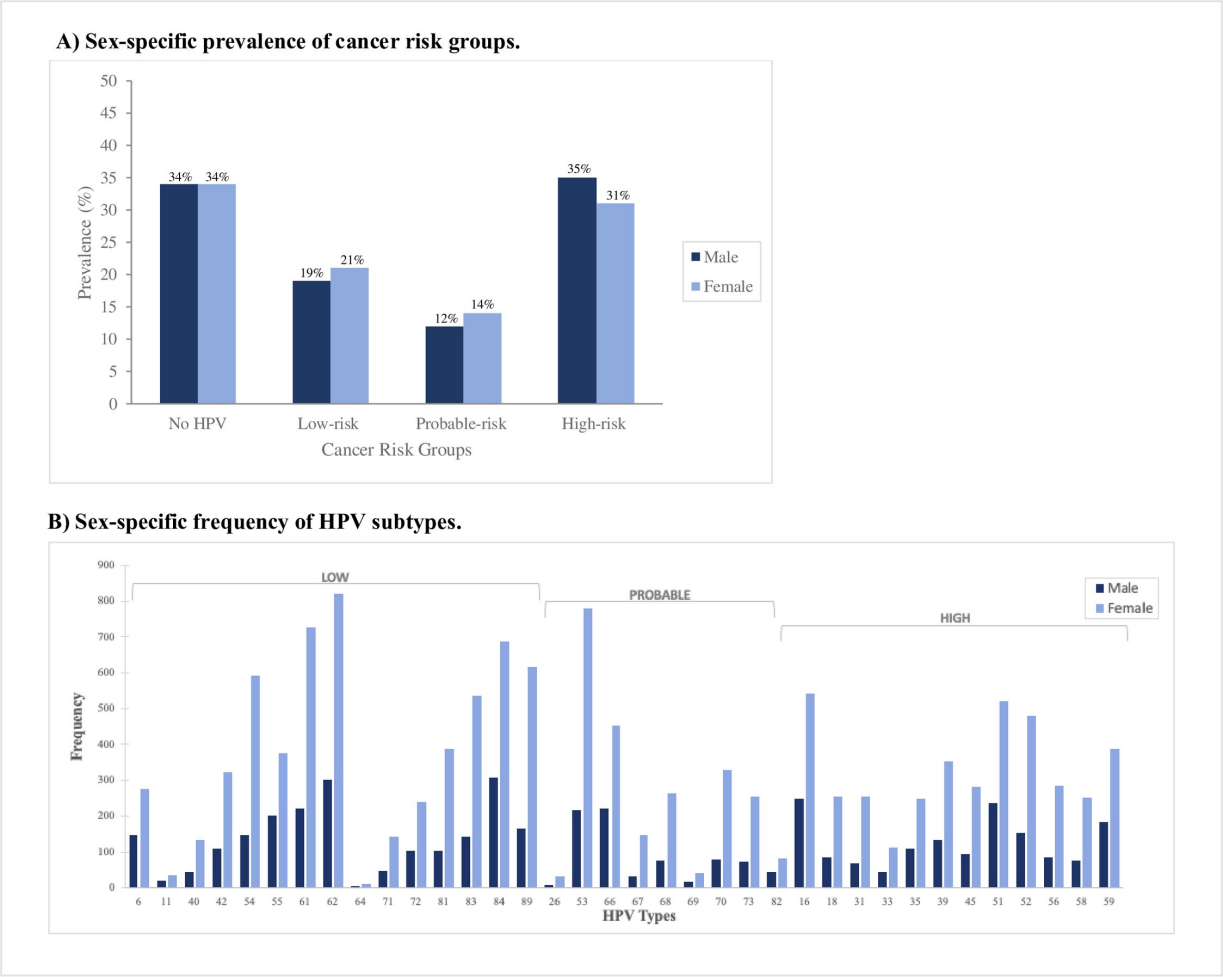
Sex-specific prevalence of cancer risk groups and frequency of HPV subtypes according to cancer risk groups. Panel B) Individuals with multiple infections of a specific cancer group appear as multiple counts if they have multiple HPV infections. For example, if an individual has an HPV type 6 and 11 infection, they will appear as a count for both bars 6 and 11. HPV testing was done through vaginal swabs for females (18-59y, 2003–2004 to 2015–2016) and penile swabs for males (18-59y, 2013–2014 to 2015–16) using Roche Linear Array Assays, and oral swabs for both males and females (18-69y, 2009–2010 to 2015–2016).

**Fig 2** Panel A shows the prevalence of each cancer risk category by sex. Overall, a majority of the sample displayed high-risk HPV (35% males, 31% females) or no HPV (34% males, 34% females). Panel B shows the unweighted frequency of HPV subtypes in the NHANES sample. Within each subtype, females had a higher case-count than males. Within the high-risk categories exclusively, ~22% of females and 22% of males displayed subtype 16, whereas 8% of males and 11% of females displayed subtype 18.

**Fig 3:**
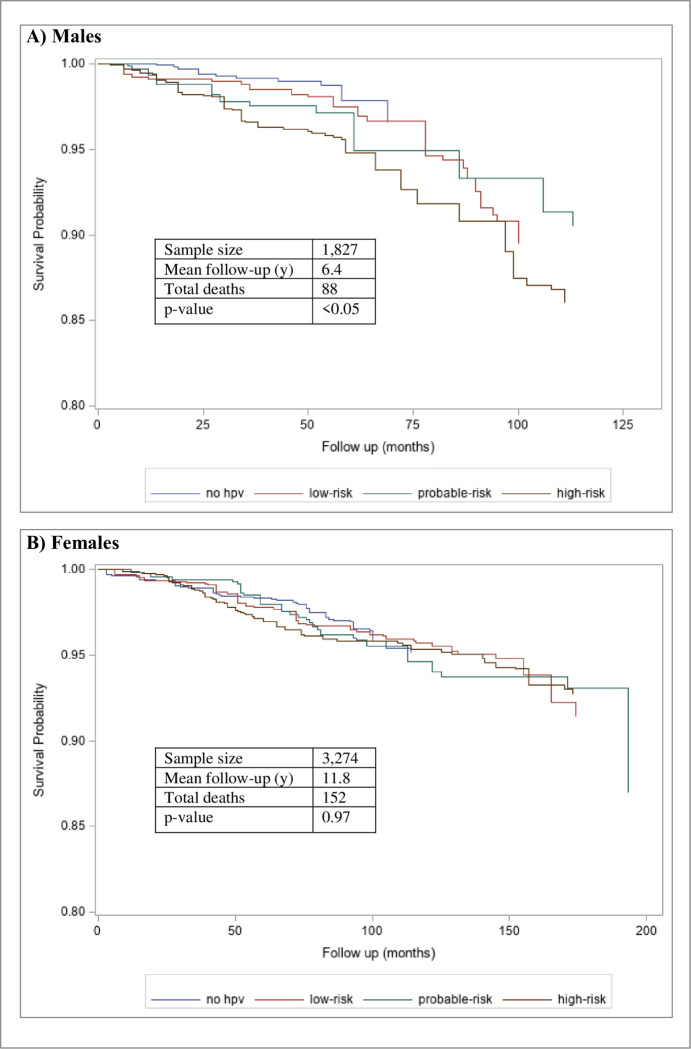
Kaplan-Meier curves across cancer risk groups, stratified by sex. Survival differences across cancer risk groups was assessed by global chi-squared analysis.

**Fig 3** displays the survival probability of the HPV risk status in men and women. Over an average 9.4 years of follow-up there were 240 all-cause deaths (no HPV: n = 46 deaths; low-risk: n = 60 deaths; probable: n = 37 deaths, and; high-risk: n = 97 deaths). Visual inspection of the survival probability curves is suggestive of lower survival probabilities among the probable-risk and high-risk HPV groups for males (p<0.05), and no clear relationships observed in females (p = 0.97).

**Table 2** shows the cross-sectional association between HPV status and MetS, stratified by sex. Compared to females with no HPV (OR = 1.00, ref), the odds of MetS were lower in those with high-risk HPV (OR = 0.74, 95% CI: 0.57–0.97), however these results were no longer significant in models that covaried for age, smoking status, health insurance, physical activity, and education. There were no significant associations between HPV status and MetS observed in males.

**Table 2:** Odds of MetS across cancer risk groups, stratified by sex.

	Male		Female	
Unadjusted	OR	95% CI	OR	95% CI
**HPV status**				
No HPV	1.00	ref	1.00	ref
Low risk HPV	1.07	0.66–1.68	1.02	0.79–1.31
Probable risk HPV	1.06	0.64–1.75	0.84	0.60–1.17
High risk HPV	0.94	0.63–1.41	0.74*	0.57–0.97
**Adjusted**				
**HPV status**				
No HPV	1.00	ref	1.00	ref
Low risk HPV	1.06	0.67–1.67	0.91	0.69–1.20
Probable risk HPV	1.04	0.64–1.69	0.80	0.59–1.10
High risk HPV	0.94	0.61–1.44	0.77	0.58–1.03

This table provides results of logistic regressions; assessing the odds of MetS across cancer risk groups in unadjusted and fully adjusted models, stratified by sex.

OR: odds ratio.

The adjusted model accounted for age, smoking status, health insurance, physical activity, and education.

*Indicates significance, p<0.05.

**Table 3** shows the association between HPV cancer risk status on risk of mortality. Relative to males with no HPV, those with high-risk HPV (HR = 2.59, 95% CI: 1.22–5.49) were at increased risk of all-cause death, however these results were no longer significant after adjustment for covariates. No significant associations between HPV status on risk of mortality were found in females.

**Table 3:** Effects of HPV on risk of mortality, stratified by sex.

	Male		Female	
Unadjusted	HR	95% CI	HR	95% CI
HPV status				
No HPV	1.00	ref	1.00	ref
Low risk HPV	1.62	0.73–3.60	1.10	0.60–2.01
Probable risk HPV	1.78	0.64–4.92	1.20	0.60–2.36
High risk HPV	2.59*	1.22–5.49	1.12	0.64–1.98
**Adjusted**				
**HPV status**				
No HPV	1.00	ref	1.00	ref
Low risk HPV	1.25	0.52–3.00	0.89	0.48–1.65
Probable risk HPV	1.55	0.54–4.43	1.05	0.54–2.06
High risk HPV	2.03	0.87–4.71	1.08	0.61–1.91

This table provides results of cox regressions; assessing the risk of mortality across cancer risk groups in unadjusted and fully adjusted models, stratified by sex.

HR: hazard ratio.

The adjusted model accounted for age, smoking status, health insurance, physical activity, and education.

*Indicates significance, p<0.05

**Fig 4:**
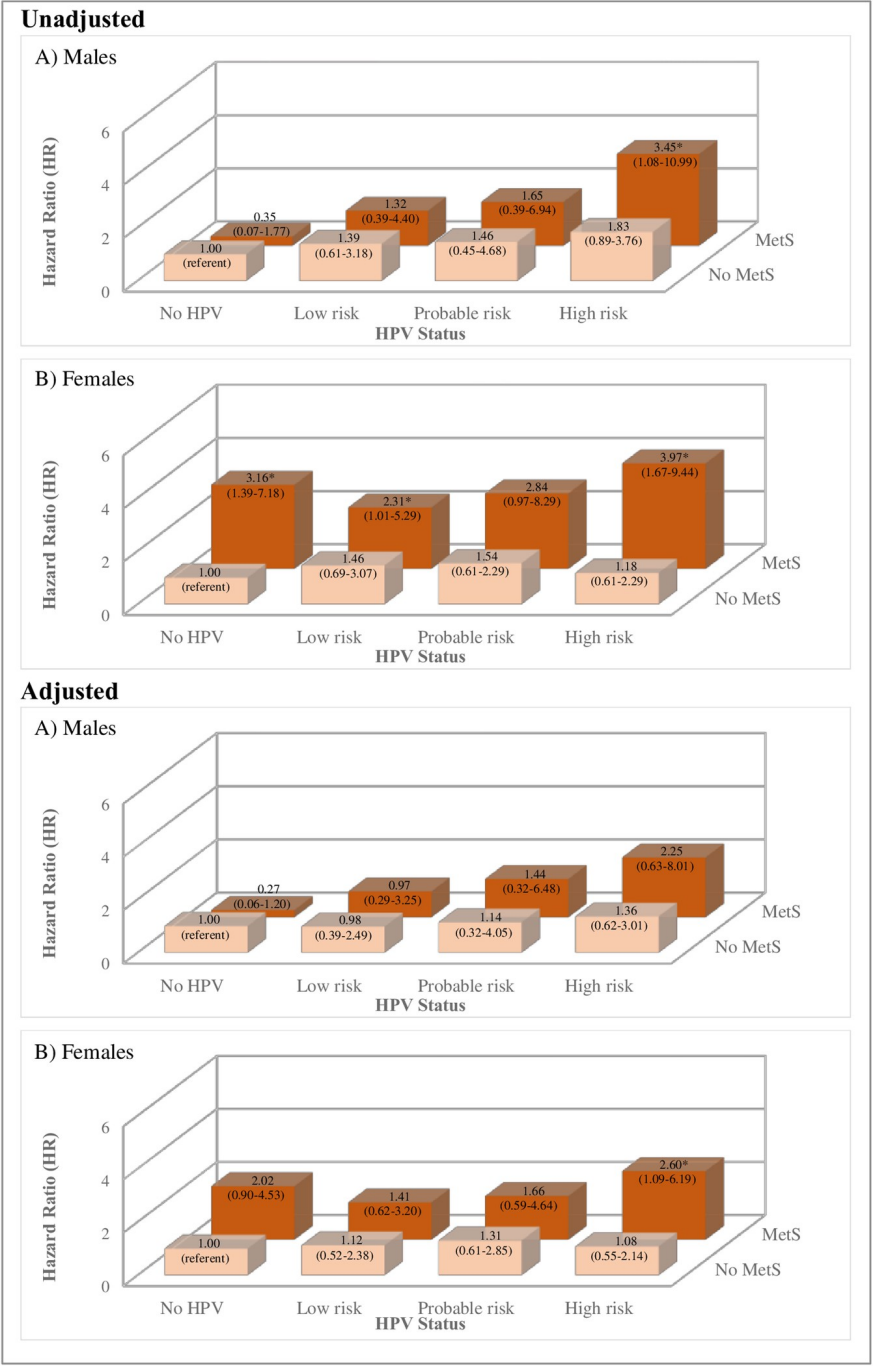
Effects of HPV and MetS on risk of mortality, stratified by sex. These figures provide results of cox regressions to assess risk of mortality across cross-classified cancer risk groups and MetS in unadjusted and fully adjusted models, across males and females. HR = hazard ratio.

Finally, **Fig 4** displays the sex-specific mortality risk across each HPV/MetS strata. In males, there was over a three-fold higher risk of mortality in the high-risk HPV/MetS groups compared to those with no HPV and no MetS; however, fully adjusted models revealed no significant associations between HPV/MetS groups and mortality in males. In females, mortality risk was four-fold higher in those with high-risk HPV/MetS compared to those with no HPV and no MetS, an effect that was moderately attenuated with further adjustment (HR = 2.60, 1.09–6.19).

## Discussion

The current study extends previous research on the risk of cancer morbidity and mortality with high-risk HPV by examining the joint effect of MetS, a common cluster of pre-clinical risk factors, and HPV sub-type on all-cause death. Using a nationally representative sample of US adults (2003–2016) with an average of 9.4 y of follow-up we observed that the co-occurrence of MetS and high-risk HPV notably elevated risk of mortality in females.

In the pooled NHANES sample, HPV types 16 and 18 accounted for approximately 22% and 10% of high-risk HPV, respectively. Consistent with previous literature [30, 31], HPV type 16 was the most common high-risk HPV subtype in both men and women. In this sample, high-risk HPV was highest among females aged 18 to 24 years old, and subsequently decreased with age. By contrast, high-risk HPV tended to increase with age in males. These findings are generally consistent with global systematic reviews that report a decrease in HPV prevalence with age in females [32], and lower prevalence of HPV subtypes of concern in younger males [33].

Our results diverge slightly from existing literature on the distribution of HPV sub-types in that we assigned HPV group risk (“none”, “low”, “probable”, and “high”) as the “highest” HPV sub-type observed within an individual. In our study, high-risk HPV sub-types were the most common; however, low-risk genotypes tended to be the most prevalent HPV sub-type [34]. Screening characteristics for co-infections within the low-risk category or multiple positive low-risk genotypes [35] may have contributed to our under-counting of low-risk HPV sub-types in our sample. Indeed, enhanced screening of low-risk cases, that would not otherwise be picked up in the general population due to no routine use of HPV testing in the U.S. for low-risk strains, could improve accuracy of predictions. At the time of the NHANES data collection primary screening was limited to those with a cervix and would not include screening for low-risk HPV. Screening for low-risk HPV status lacks clinical utility; as such, knowing low-risk HPV status, or knowing that a low-risk HPV strain is present may not necessarily have an impact on the clinical management of patient with non-malignant conditions such as mucosal warts. In contrast, current screening tailors to the clinical utility of knowing high risk HPV status as it has an impact on the treatment of precancerous lesions and the prevention of cancer.

Previous literature has examined the co-occurrence of MetS and HPV and risk of HPV persistence [15, 16], as well as HPV-related cancers and conditions [17–25]. Findings from these studies indicate that MetS is associated with a greater risk of HPV persistence [15, 16], and that MetS or its individual components tend to increase the risk of cancers related to HPV [17–25]. Adding to the literature, we examined MetS risk across HPV cancer-risk groups, and mortality risk across cross-classified groups of MetS and HPV. In these analyses, females with high-risk HPV had lower odds of MetS relative to the group with no HPV, and there were no associations between HPV and MetS observed in males. After adjusting for age, smoking status, health insurance, physical activity, and education, having MetS and high-risk HPV increased the risk of mortality in females.

While there are several possible explanations, these findings may be due in part to sex differences in vaccination and screening. Indeed, the Centers for Disease Control and Prevention (CDC) have noted sex differences in HPV vaccination from 2013–2018 wherein females were more likely to have ever received one or more dose of HPV vaccine compared to males [36]. In 2014, 60% of females and only 42% of males aged 13 to 17 years old received at least one dose of the HPV vaccine [37]. There are also disparities in HPV-related cancer screening. Beginning at the age of 21, females are advised to undergo cytology (pap) test [38] to detect precancerous cell changes that could lead to cervical cancer. To date, there is no routine HPV-related cancer screening guidelines in place for males. Variations in NHANES HPV testing methodology might account for some observed disparities in mortality across sex. Females were assessed by oral swabs for four cycles (18-69y) and vaginal swabs for seven cycles (18-59y), whereas males were only assessed by penile swabs for two cycles (18-59y) and oral swabs for four cycles (18-69y). Consequently, men aged 60–64 were only evaluated for HPV in two cycles, potentially leading to an underestimation of mortality.

The exact mechanism of association between HPV and MetS remains unclear, but may be related to a persistent inflammatory response and increased oxidative stress [39]. Thus, MetS could put an individual at a higher risk of HPV-related cancers, which pose a higher risk of mortality when co-occurring with an HPV infection. Previous literature has also found elevated mortality risk in HPV-related cancers [26, 27], but mortality risk in cancer risk groups remains unclear. Mortality findings in this study indicate greatest risk of mortality in the high-risk HPV groups relative to the group with no HPV, in females. A significant proportion (70%) of cervical cancers are associated with high-risk HPV, specifically type 16 and 18 HPV [9], therefore our findings of elevated mortality risk in high-risk HPV groups was expected.

## Strengths and limitations

Among several strengths of the current analysis is the use of NHANES data which allows for nationally representative estimates using comprehensive health behavior and laboratory information. This dataset is unique in that it captures HPV and MetS variables from objective laboratory data and allows for the mutual adjustment of these factors. The main limitation of this study is a lack of information on HPV persistence (a pre-cursor for cancer), as HPV testing was conducted in NHANES laboratories only once per participant. Furthermore, the prevalence of HPV in males may be underestimated, as females were assessed by oral swabs for four cycles (2009–2016) and vaginal swabs for seven cycles (2003–2016), whereas males were only assessed by penile swabs for two cycles (2013–2016) oral swabs for four cycles (2009–2016). The prevalence of detectable HPV has shown to be higher through penile swabs (45%) than oral swabs (11%) in US male adults [40]. Because the no HPV group had a relatively shorter average follow-up than the low, probable, and high-risk groups, we can not exclude the possibility that further screening would have resulted in a classification as HPV positive. This, however, would have resulted in a bias to the null in the current analysis. Finally, the analytical sample excluded individuals with missing data on HPV, MetS, mortality, or covariates, which may be reflective of treatment seeking behaviors seen in other studies.

## Conclusion

Taken together, results from this study demonstrate the importance of a common cluster of cardiovascular risk factors on mortality risk across HPV subgroups. Future efforts focused on the harmonization of HPV-specific datasets or pooling of subsequent NHANES cycles may allow for broader insight into this question, by examining specific HPV subtypes, highly prevalent high-risk HPV subtypes, and HPV-related cancers. In the intermediate term, further prospective analysis is needed to understand the temporal, age, vaccination, and sex effects of HPV diagnosis on these relationships in studies with more detailed histories of HPV infection and persistence.

## Supporting information

S1 Checklist (DOCX)
